# Induction of Erythroid Differentiation in Human Erythroleukemia Cells by Depletion of Malic Enzyme 2

**DOI:** 10.1371/journal.pone.0012520

**Published:** 2010-09-02

**Authors:** Jian-Guo Ren, Pankaj Seth, Peter Everett, Clary B. Clish, Vikas P. Sukhatme

**Affiliations:** 1 Divisions of Interdisciplinary Medicine and Biotechnology, Hematology-Oncology and Nephrology, Beth Israel Deaconess Medical Center (BIDMC) and Harvard Medical School, Boston, Massachusetts, United States of America; 2 Metabolite Profiling Initiative, The Broad Institute of the Massachusetts Institute of Technology and Harvard, Cambridge, Massachusetts, United States of America; Texas A&M University, United States of America

## Abstract

Malic enzyme 2 (ME2) is a mitochondrial enzyme that catalyzes the conversion of malate to pyruvate and CO_2_ and uses NAD as a cofactor. Higher expression of this enzyme correlates with the degree of cell de-differentiation. We found that ME2 is expressed in K562 erythroleukemia cells, in which a number of agents have been found to induce differentiation either along the erythroid or the myeloid lineage. We found that knockdown of ME2 led to diminished proliferation of tumor cells and increased apoptosis *in vitro*. These findings were accompanied by differentiation of K562 cells along the erythroid lineage, as confirmed by staining for glycophorin A and hemoglobin production. ME2 knockdown also totally abolished growth of K562 cells in nude mice. Increased ROS levels, likely reflecting increased mitochondrial production, and a decreased NADPH/NADP^+^ ratio were noted but use of a free radical scavenger to decrease inhibition of ROS levels did not reverse the differentiation or apoptotic phenotype, suggesting that ROS production is not causally involved in the resultant phenotype. As might be expected, depletion of ME2 induced an increase in the NAD^+^/NADH ratio and ATP levels fell significantly. Inhibition of the malate-aspartate shuttle was insufficient to induce K562 differentiation. We also examined several intracellular signaling pathways and expression of transcription factors and intermediate filament proteins whose expression is known to be modulated during erythroid differentiation in K562 cells. We found that silencing of ME2 leads to phospho-ERK1/2 inhibition, phospho-AKT activation, increased GATA-1 expression and diminished vimentin expression. Metabolomic analysis, conducted to gain insight into intermediary metabolic pathways that ME2 knockdown might affect, showed that ME2 depletion resulted in high orotate levels, suggesting potential impairment of pyrimidine metabolism. Collectively our data point to ME2 as a potentially novel metabolic target for leukemia therapy.

## Introduction

Malic enzymes (ME; EC 1.1.1.40) represent a family of oxidative decarboxylases that catalyze the divalent metal ion (Mn^2+^ or Mg^2+^) dependent irreversible oxidative decarboxylation of L-malate to yield CO_2_ and pyruvate, with concomitant reduction of dinucleotide cofactor NAD^+^ or NADP^+^ [1]. In different species, these enzymes show highly conserved sequences and similar overall structural topology, suggesting important biological functions, which however are not well-defined. Three isoforms of malic enzyme have been identified in mammals according to their nucleotide specificity: cytosolic NADP^+^-dependent (ME1), mitochondrial NAD^+^-dependent (ME2), and mitochondrial NAD(P)^+^-dependent malic enzyme (ME3). It has been shown that ME2 favors NAD^+^ as a cofactor under physiological conditions, although it can use both NAD^+^ and NADP^+^. ME2 is thought to be involved in the ultimate conversion of malate to citrate, the latter being directed to the cytosol *via* the carboxylate carrier [2]. Citrate that is extruded from mitochondria is the primary precursor for the endogenous synthesis of fatty acids, cholesterol, isoprenoid, as well as acetylation reactions that modify proteins.

There is a paucity of information on the role of malic enzymes in normal physiology and in disease states. It is important to recognize that the literature is confusing in that sometimes the malic enzyme is not specified (i.e. ME1 vs 2 vs 3) and in some cases, the enzyme is confused with two of the malate dehydrogenases. Thus we cite here only references that provide the EC number for ME2 or that clearly specify the activity in question as being due to a mitochondrial NAD^+^ dependent enzyme that produces pyruvate from malate. Previous studies indicate that both the cytosolic and mitochondrial malic enzyme are involved in malate-pyruvate cycling under conditions of nutrient-stimuated insulin secretion [3], [4], [5]. In neurons pyruvate produced from malate is a substrate for the neuronal synthesis of γ-aminobutyric acid, found to be associated to idiopathic generalized epilepsy through linkage analysis [6]. ME2 gene expression is 5.6-fold lower in anterior cingulate tissue from post-mortem bipolar brains, associated with both psychotic and manic disorders including schizophrenia and bipolar disorders [6], [7]. From a cancer standpoint, ME2 activity increases with progression to neoplasia in a rat tracheal epithelial line [8] with similar findings in Morris hepatomas [9]. Of interest, ME2 is present in tumor mitochondria in levels proportional to the rate of cell division [9], [10], while curiously it is essentially absent in liver, regenerating liver, and the mitochondria of other organs. ME2 interacts directly with the malate-aspartate shuttle system. It is believed that ME2, via the generation of NADH and pyruvate products, may play an important role in the metabolism of glutamine, which is needed to produce both reducing equivalents and energy in rapidly proliferating tissues such as tumors. ME2 is regulated by ATP which acts as an inhibitor and by fumarate which acts as an activator of its catalytic activity [11], [12], [13]. This regulation is consistent with the functional role of this enzyme, as ATP is an overall product of energy metabolism and fumarate is generated by the previous step in the TCA cycle. The possible functional involvement of ME2 in neoplasia is strongly suggested by its increased activity in tumor tissue. This finding implicates the enzyme as a potentially attractive novel anti-cancer target and warrants detailed investigation of its functional role in cancer.

The human K562 erythroleukemia cell line is a multipotent hematopoietic precursor cell line derived from a patient with chronic myeloid leukemia (CML) in blast crisis and thus provides a model system to study gene expression during hematopoiesis. These cells can be induced to differentiate along either the erythroid or megakaryocytic lineages. A variety of chemical compounds, such as hemin, butyrate, cisplatin, PMA, TPA, Ara-C, the BCR-ABL signaling inhibitor imatinib, and the Hsp90 inhibitor radicicol have been found to induce K562 differentiation [14], [15]. Here we have used the K562 model system to study the function of ME2 and find that silencing this gene leads to K562 cell apoptosis and erythroid differentiation and abolishes growth of these cells in vivo.

## Results

### Knockdown of endogenous ME2 levels by shRNA impairs proliferation of K562 cells

**Figure 1:**
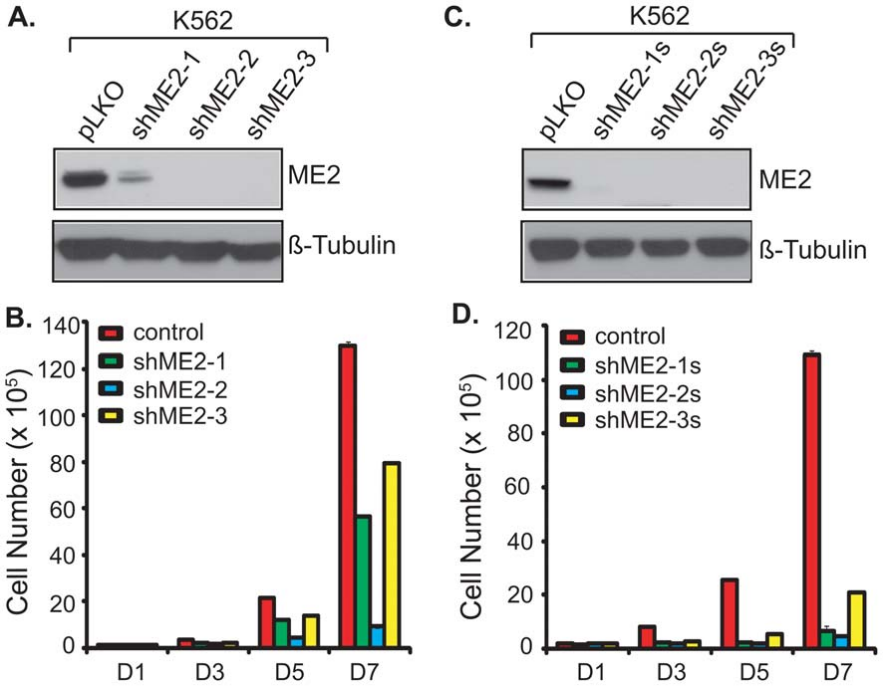
Effects on K562 cell proliferation of three independent shRNA hairpins targeting ME2. A: Western blot analysis using an ME2 antibody of lysate from respective pools of cells transduced with three independent ME2 shRNA lentiviruses, and following selection of puromycin for 10 days as described under “Materials and Methods”. Data are representative of two independent experiments. All three pools showed marked ME2 silencing. B: Cell proliferation in K562 cells transduced with the indicated shRNA lentiviral constructs as described in “A”. Data are representative of three independent experiments. C: Western blot analysis of cellular extracts in single clone K562 cells as described under “Materials and Methods” demonstrated effective knockdown of ME2 levels. Data are representative of two independent experiments. D: Cell proliferation of K562 single cell clones with ME2 knockdown derived from the corresponding pools as described in “C”. Data are representative of three independent experiments.

To investigate the effects of ME2 inhibition on K562 cells, we produced recombinant lentiviral particles by expressing constructs containing ME2 short hairpin RNAs (shRNA) and established stable cell clones. Three independent constructs, shME2-1, shME2-2, and shME2-3, were used to generate stable cell lines. Each of these pools displayed significantly reduced ME2 protein levels compared to control lentiviral vector only (pLKO) infected cells (Figure 1A). These three pools were used in experiments described in the Methods section to examine the role of ME2 in K562 cell proliferation. As shown in Figure 1B, stable lentiviral reduction of ME2 protein levels resulted in dramatic inhibition of K562 cell growth *in vitro*. Furthermore, we established three single clones from the three independent pools, designated as shME2-1s, shME2-2s, and shME2-3s. The three single clones also displayed marked reduction in ME2 protein levels (Figure 1C). As anticipated, cell proliferation was markedly decreased in these cells (Figure 1D).

### Stable knockdown of endogenous ME2 levels by shRNA induces erythroid differentiation in K562 cells

**Figure 2:**
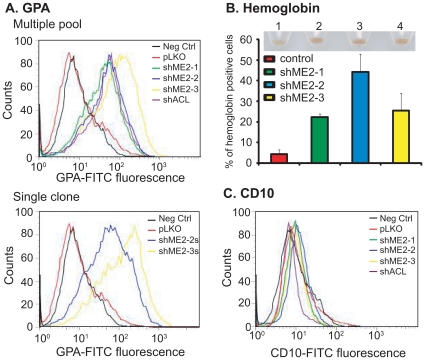
Stable knockdown of endogenous ME2 levels in K562 cells induces erythroid differentiation. A: Expression levels of the erythroid marker glycophorin A on the surface of control (pLKO) and ME2 knockdown cells (shME2-1, shME2-2 and shME2-3) were compared using a mouse FITC-conjugated anti-human glycophorin A antibody. As a negative control (Neg Ctrl), cells were incubated with FITC-conjugated control IgG. As a positive control, K562 cells were transduced with ATP citrate lyase (ACL) shRNA lentiviral particles (ACL inhibition is known to cause erythroid differentiation in K562 cells – referenced in the text), and incubated with mouse FITC-conjugated anti-human glycophorin A antibody. The control clone was generated by stable transduction of control pLKO vector, while clones shME2-1, shME2-2 and shME2-3 were generated using the pLKO-ME2 shRNA lentivirus. Data are representative of three independent experiments. B: The percentage of hemoglobin-expressing cells in control (pLKO) and ME2 knockdown (shME2-1, shME2-2 and shME2-3) cell populations was determined by benzedrine staining. Plotted is the mean ± SD from triplicate samples from a representative experiment. Insert: cell pellets from ME2 knockdown cells. 1: pLKO; 2: shME2-1; 3: shME2-2; 4: shME2-3. Increased brown color is clearly visible in lanes 2, 3 and 4. C: Expression levels of the megakaryocytic marker CD10 on the surface of control (pLKO) and ME2 knockdown cells (shME2-1, shME2-2 and shME2-3) were compared using a mouse FITC-conjugated anti-human CD10 antibody. As a negative control (Neg Ctrl), cells were incubated with FITC-conjugated control IgG. The control clone was generated by stable transduction of control pLKO vector, while clones shME2-1, shME2-2 and shME2-3 were generated using the pLKO-ME2 shRNA let virus. Data are representative of three independent experiments.

Next, we asked whether ME2 silencing would lead to differentiation of K562 cells. We assayed for erythroid differentiation by analyzing our pooled ME2 knockdown cells for expression of glycophorin A (CD235a), a cell surface glycoprotein expressed selectively on erythroid precursors and mature erythrocytes. Previously it has been shown that glycophorin A is induced in K562 cells undergoing erythroid differentiation in response to imatinib [16] or radicicol [15]. Compared to vector transduced cells, ME2 knockdown cells displayed an increase in the surface expression of this erythroid cell marker (Figure 2A). Similar results were also obtained in single clones derived from shME2-2 and shME2-3 pools (Figure 2B). Furthermore, quantitation of hemoglobin-positive cells revealed that approximately 21.2%, 50.32%, and 31.52% of cells within the shME2-1, shME2-2 and shME2-3 ME2 knockdown population, respectively, expressed hemoglobin, compared to 4.4% for vector control infected K562 cells (Figure 2B and Figure S1).

K562 cells can also differentiate along the megakaryocytic lineage. We assessed our ME2 knockdown cells for their degree of megakaryocytic differentiation by analyzing the cells for expression of CD10, a cell surface glycoprotein expressed selectively on mature megakaryocytic cells that is induced in K562 cells in response to PMA stimulation. As shown in Figure 2C, there was no difference in expression CD10 between control and ME2 knockdown cells, suggesting that deficiency of ME2 cells directs K562 cells toward erythroid differentiation.

### Knockdown of endogenous ME2 leads to apoptosis in K562 cells and suppresses tumor growth *in vivo*

**Figure 3:**
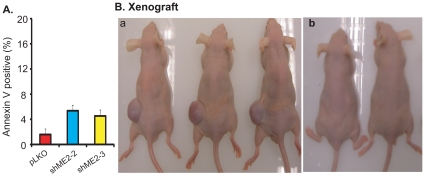
Stable knockdown of endogenous ME2 levels in K562 cells results in apoptosis *in vitro* and suppresses tumor formation from K562 cells *in vivo*. A: Knockdown of ME2 induces apoptosis in K562 as detected using the annexin V reagent. Data are expressed as mean ± SD, n = 3. B: Stable knockdown of ME2 in K562 cells failed to generate tumors in nude mice. Approximately 10^7^ ME2 deficient or control K562 cells resuspended in 200 µl of a serum-free culture medium/Matrigel mixture (1∶1) were subcutaneously implanted into female athymic nude mice as described under “Materials and Methods”. Tumor-bearing mice were sacrificed after 4 weeks and the mice photographed before excision and weighing. a, Left (L): pLKO; Right (R): shME2-2: b, L and R: shME2-3. Tumors formed only in the pLKO transduced cells.

Tumor cell differentiation is often accompanied by increased apoptosis [17]. Therefore, we examined apoptosis in K562 cells with ME2 depletion by annexin-V/7-AAD staining. As shown in Figure 3A, knockdown of ME2 levels caused a 2.8 to 3.3-fold increase in basal apoptosis.

Next, we asked what the effects of ME2 depletion would be *in vivo* by examining tumorigenicity of K562 cells in mice. We injected vector control and ME knockdown clones (shME2-2 and shME2-3) subcutaneously into nude mice and assessed tumor formation and progression following injections. In one group of 4 mice, each mouse received a vector control inoculation in one flank and an ME2 shRNA clone in the other, so that tumor comparisons would be controlled for each individual mouse. In another group of 2 mice, both of the flanks were injected with another ME2 shRNA clone. Growth of tumors was monitored weekly, and tumors were excised and weighed 6 to 8 weeks postinjection. The K562 ME2 knockdown cells failed to form tumors (Figure 3B).

### Depletion of endogenous ME2 levels enhances reactive oxygen species (ROS), increases NAD^+^/NADH and NADP^+^/NADPH ratio and inhibits ATP production in K562 cells

The role of ROS in cell differentiation and cell survival is complex. There is some evidence supporting the notion that certain cancer cells are under increased oxidant stress. In particular, increased ROS generation occurs in cancer cells carrying mutations or activation of Ras, Bcr-Abl, and c-Myc [18], [19], [20]. Further increases in ROS can promote differentiation and additional increases can lead to apoptosis. Indeed, anticancer drugs including doxorubicin, arsenic trioxide, and taxol have been observed to generate ROS. Increasing ROS can induce cell differentiation in K562 cells and in neuroblastoma cells [21], [22], [23].

**Figure 4:**
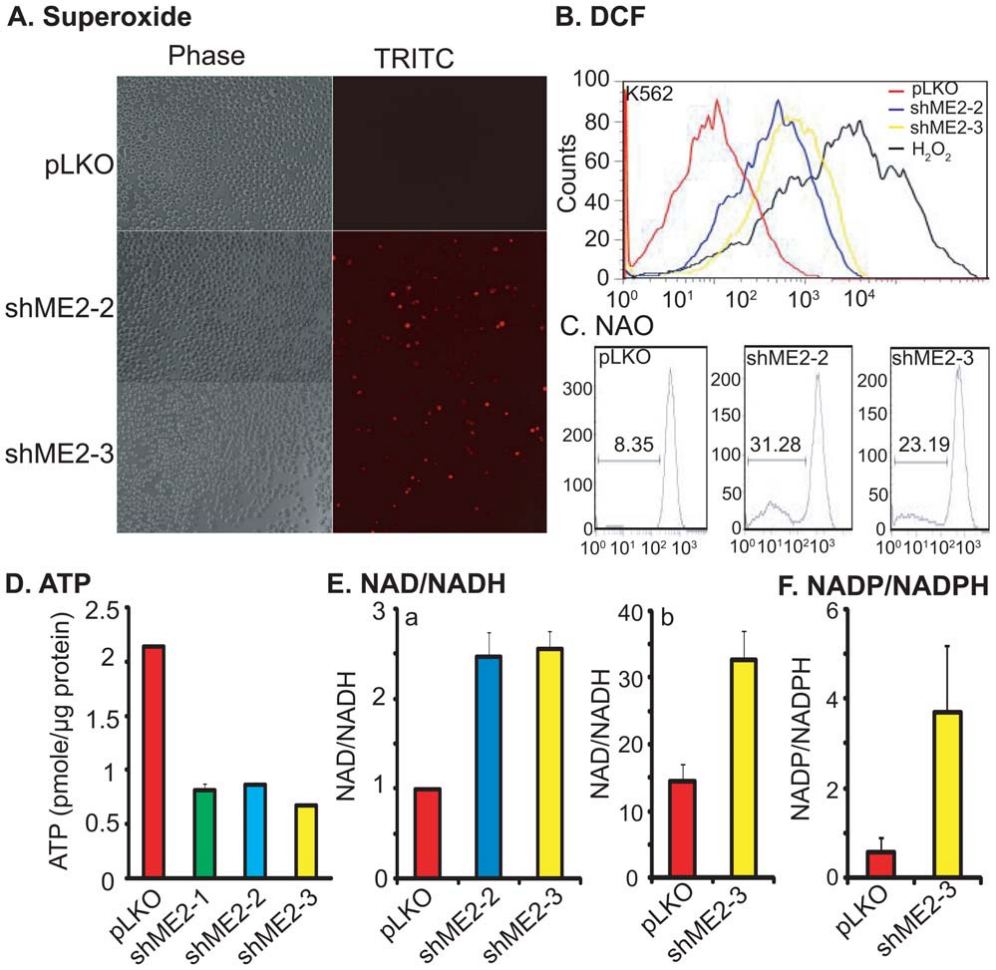
Depletion of endogenous ME2 enhances ROS generation, increases NAD^+^/NADH and NADP/NADPH ratios and decreases ATP levels. A: Accumulation of mitochondrially generated superoxide in K562 ME2 knockdown cells as detected by MitoSOX. Data are representative of two independent experiments. B: Increased ROS in K562 ME2 knockdown cells detected by flow cytometry using CM-H_2_DCF-DA. Each histogram is representative of three experiments. C. Comparison of oxidative damage to cardiolipin in ME2 knockdown versus control K562 cells. M1 indicates subpopulation of cells that lost NAO signal due to cardiolipin oxidation. D. Depletion of ME2 inhibits ATP production in K562 cells. Data are expressed as mean ± SD, n = 3. E: Depletion of ME2 increases NAD^+^/NADH ratio. a, NAD^+^ and NADH were measured by NAD/NADH Assay Kit (Abcam, San Francisco, CA) as described in “Materials and Methods”. Data are expressed as mean ± SD, n = 3. b, NAD+ and NADH were measured by LC-MS methods as described in “Materials and Methods”. F. Depletion of ME2 increases NADP/NADPH ratio in ME2 knockdown cells. NADP and NADPH were measured by LC-MS methods as described in “Materials and Methods”.

To determine if knockdown of ME2 promotes ROS production, we first analyzed superoxide production in K562 cells with ME2 depletion by using the superoxide detection reagent MitoSOX Red, a novel fluorogenic dye for highly selective detection of superoxide in the mitochondria of live cells. This reagent is live-cell permeant and is rapidly and selectively targeted to the mitochondria. Once in the mitochondria, it is rapidly oxidized by superoxide but not by other ROS- or reactive nitrogen species-generating systems. We therefore stained ME2 knockdown K562 cells with MitoSOX Red, and observed the cells under fluorescence microscopy. We only found positive cells in ME2 deficient K562 cells as compared to controls (Figure 4A). There was a significant increase in MitoSOX red fluorescence in ME2 knockdown cells indicative of an increase in superoxide generation. The stability of superoxide is short-lived in cells, since superoxide dismutase rapidly converts it into H_2_O_2_. Therefore, we further assayed for H_2_O_2_ and other reactive species in response to ME2 knockdown as quantified by flow cytometry using CD-H_2_DCF-DA as a fluorescent probe. Compared to empty vector control, we observed a significant increase in basal ROS content in three independent ME2 shRNA knockdown cells (Figure 4B). Furthermore, we detected oxidation of cardiolipin, a mitochondrial membrane lipid component by labeling with NAO. As shown in Figure 4C, knockdown of ME2 caused massive cardiolipin oxidation. About 31.28% and 23.19% of ME2 depleted cells showed cardiolipin oxidation in two independent clones, respectively (Figure 4C). Moreover, intracellular NADPH level has been considered as an effective antioxidant. In agreement with the increase of ROS in K562 cells, we found NADPH level decreased in ME2 knockdown cells, a result that was confirmed using metabolomic analysis described above (Figure 4F).

ME2 may play an important role in energy production fueled by glutamine. Therefore, we examined ATP production in cells with ME2 deficiency. As illustrated in Figure 4D, knockdown of ME2 led to almost 50% ATP inhibition in three independent ME2 shRNA cells. Consistent with this observation, we noted a 2-fold increase in the NAD^+^/NADH ratio in ME2 knockdown cells (Figure 4E).

### Inhibition of ROS does not rescue ME2 deficiency induced differentiation

**Figure 5:**
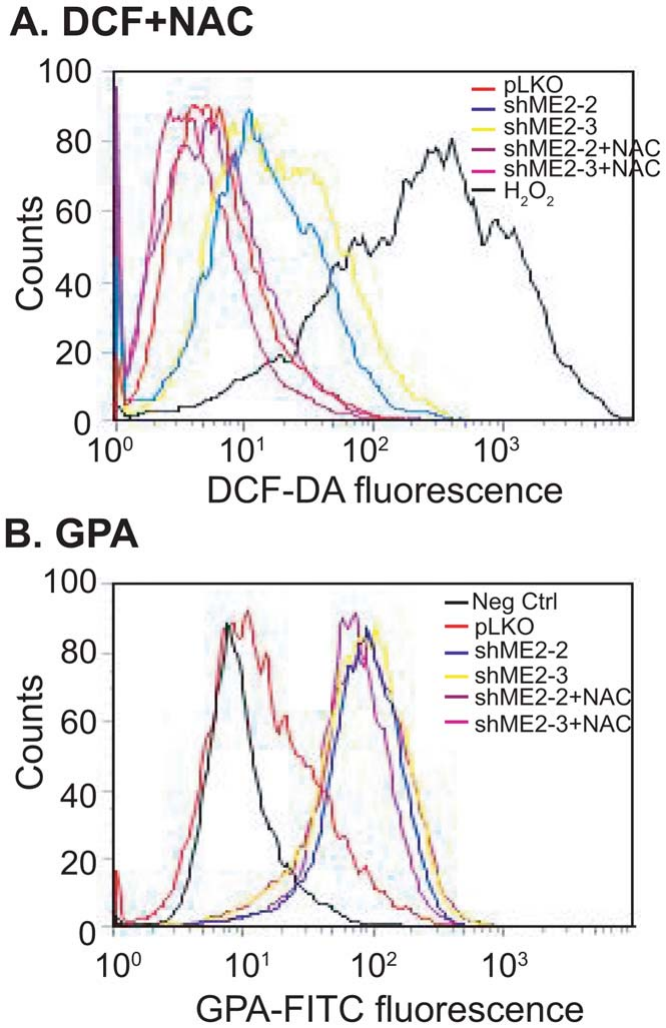
The antioxidant NAC cannot rescue ME2 knockdown induced erythroid differentiation in K562 cells. A: 5 mM NAC completely rescues ROS generation in K562 cells as detected by flow cytometry using CM-H_2_DCF-DA. Each histogram is representative of three experiments. B: ROS inhibition by 5.0 mM NAC did not rescue ME2 knockdown induced erythroid differentiation in K562 cells. Each histogram is representative of three experiments.

ROS have been shown to induce K562 cell differentiation *in vitro* [22]. We asked whether differentiation induced by knockdown of ME2 occurs via increased ROS generation. To answer this question, we inhibited ROS using NAC and examined differentiation in K562 leukemia cells induced by ME2 depletion. 5.0 mM NAC completely inhibited endogenous ROS in ME2 knockdown leukemia cells (Figure 5A), without affecting differentiation (Figure 5B), suggesting that ROS production is only a by-product of ME2 knockdown.

### The malate-aspartate shuttle (MAS) inhibitor amino-oxyacetate (AOA) cannot induce K562 differentiation but induces cell death

**Figure 6:**
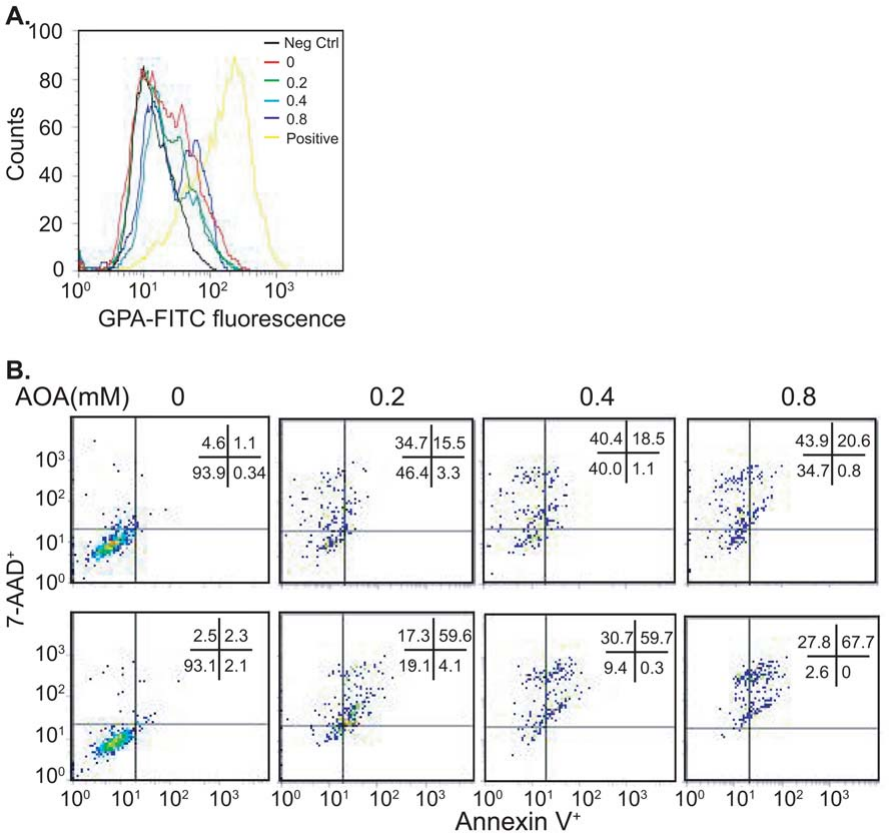
Supplementation by exogenous amino-oxyacetate in medium cannot induce erythroid differentiation but does induce cell death. A. Cells were treated with different concentrations of amino-oxyacetate and expression levels of the erythroid marker glycophorin A on the surface of K562 cells were assessed using a mouse FITC-conjugated anti-human glycophorin A antibody. Each histogram is representative of three experiments. **B.** K562 cells with or without ME2 knockdown were incubated with different concentrations of AOA for 72 h. Cell death was assessed by flow cytometry. Top: pLKO; Bottom: shME2-3. Data are representative of two independent experiments.

One of the functions of ME2 is to generate NADH in the mitochondria. We wondered if the ME2 knockdown phenotype (K562 differentiation) might be due to decreased production of mitochondrial NADH. Since the primary function of the MAS shuttle is to transport malate into mitochondria and in so doing essentially transfer cytosolic NADH equivalents into mitochondria [24], we hypothesized that blockade of the MAS might cause differentiation. To test this hypothesis, we used AOA, which when added exogenously, is known to inhibit the aminotransferase in the MAS. As illustrated in Fig. 6A, AOA from 0.2 to 0.8 mM in the medium could not induce erythroid differentiation in K562 cells as assessed by glycophorin staining.

Next, we hypothesized that blockade of the MAS in ME2 depleted cells might lead to enhanced cell death, perhaps by further decreases in mitochondrial NADH. Though no differentiation was observed in AOA treated leukemia cells (Figure 6A), the response of ME2 knockdown and control cells to AOA treatment was quite dramatic. AOA induced leukemia cell death (mainly apoptotic) when ME2 was silenced (Figure 6B, bottom). In wild type K562 cells, AOA induced leukemia cell death (mainly necrotic) (Figure 6B, top). Interestingly, α-ketoglutarate (α-KG) could rescue AOA mediated cell death (Figure S2) suggesting that the effect of AOA was likely due to blockade of glutamate to α-KG, conversion, since AOA is known to be a nonspecific inhibitor of pyridoxal phosphate-utilizing enzymes.

### ME2 knockdown-induces K562 differentiation via the PI3K/AKT pathway

A large body of evidence indicates that the erythroid differentiation is accompanied by activation of the PI3K/AKT pathway, while megakaryocytic differentiation is accompanied by activation of the MAPK kinase pathway [25], [26], [27], [28]. In the case of erythroid differentiation, activation of PI3K/AKT is not merely an association, since blockade of this pathway inhibits differentiation [26], [27], [28].

**Figure 7:**
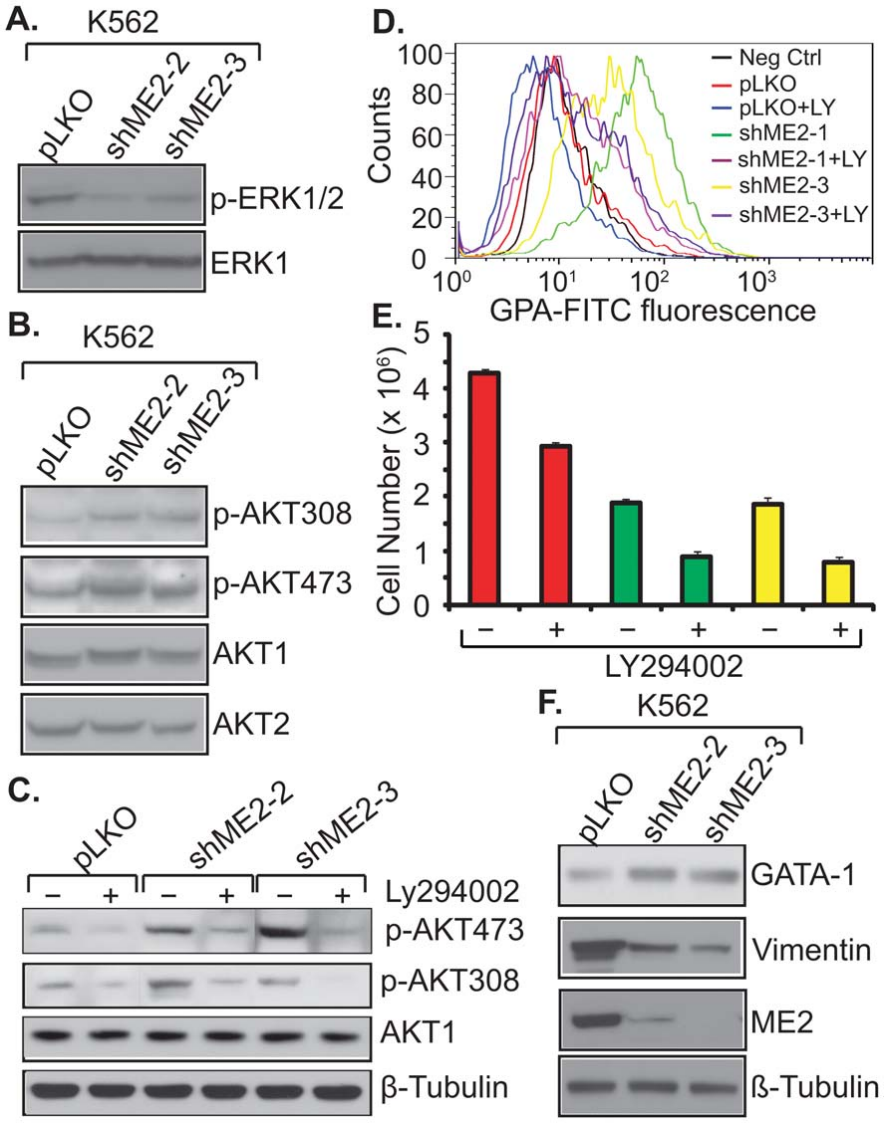
Effects of ME2 knockdown on signaling pathways, and GATA-1 and vimentin expression. K562 cells with or without ME2 knockdown were lysed with RIPA lysis buffer (50 mM Tris-HCl, pH 8.0, 150 mM NaCl, 1% Triton X-100, 1 mM EGTA) containing 1 mM PMSF and a protease inhibitor cocktail and subjected to centrifugation at 15,000× *g* for 10 min at 4°C to remove debris. After lysis, equal aliquots of protein lysate were resolved by Western blotting. Western blots were probed with anti-phospho-ERK1/2, anti-ERK1, anti-p-AKT308, anti-AKT472, anti-AKT1/2, anti-GATA-1, anti-vimentin and anti-β-tubulin. A, phospho-ERK1/2 activity in ME2 knockdown K562 cells. B, Phospho-AKT detection in ME2 knockdown K562 cells. C, 10 µM PI3K inhibitor, LY294002, inhibits p-AKT activity. D, LY294002 rescue of differentiation in ME2 knockdown K562 cells. E, The effect of LY294002 on the proliferation of K562 cells with or without ME2 knockdown. F, The expression difference of GATA-1 and vimentin in ME2 knockdown cells. Data are representative of three independent experiments.

We examined changes in these two pathways in ME2 depletion-induced differentiation. Knockdown of ME2 using two independent shRNAs resulted in a decrease in phospho-ERK levels (Figure 7A). In contrast, the phospho-AKT308 and phospho-AKT473 levels increased (Figure 7B). Furthermore, LY294002, a PI3 kinase inhibitor, which has been shown to inhibit AKT activity, rescues the differentiation mediated by ME2 silencing (Figure 7C, D, and E). These data are consistent with the literature [25], [26], [27]. GATA-1 is a transcription factor that is required and sufficient to induce erythroid differentiation [29]. We observed a GATA-1 increase in ME2 knockdown cells (Figure 7F). Vimentin expression is diminshed during erythroid differentiation [30]. In this study we found that vimentin was dramatically decreased in ME2 depleted cells (Figure 7F). Our data suggest that the differentiation induced by ME2 knockdown is most likely a PI3K/AKT regulated event which also includes ERK repression, the activation of GATA-1 transcription, and diminished expression of vimentin.

### Knockdown of endogenous ME2 levels impairs pyrimidine metabolism in K562 cells

**Figure 8:**
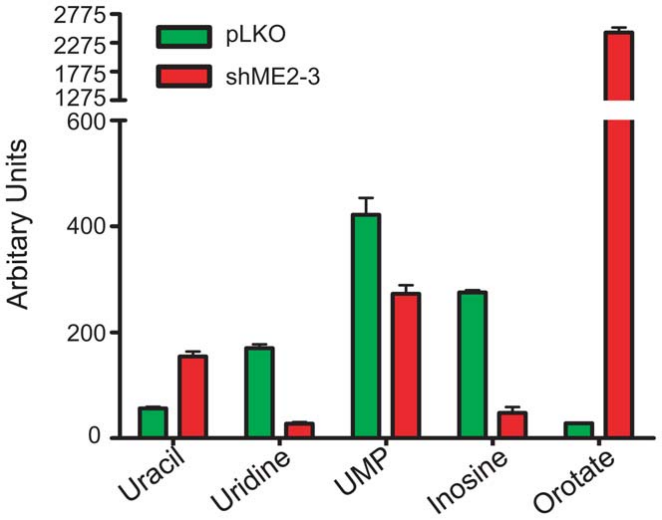
Knockdown of ME2 alters pyrimidine metabolism in K562 cells. The metabolites were measured by LC-MS method as described in “Materials and Methods”.

Several reports indicate that impairment of pyrimidine metabolism induces leukemia cells differentiation [31]. Interestingly, in our metabolomic analysis of ME2 knockdown cells we noted that there was a marked increase in the metabolite orotate (approximately 90-fold) in ME2 knockdown cells when compared to controls (Figure 8 and Table S1). Orotate is an intermediate in pyrimidine *de novo* synthesis. Its accumulation in ME2 deficient K562 cells is suggestive of a block in the synthesis or activity of UMP synthase. In support of this hypothesis, we also found modest decreases in UMP, though we are not able to explain the lower levels of uridine, cytidine and inosine (the latter involved in purinergic pathways) in ME2 knockdown cells when compared with controls (Figure 8 and Table S1).

We might have expected that orotate accumulation would lead to a block in DNA synthesis and S phase progression. However, cell cycle analysis of ME2 knockdown cells did not indicate a block in late G1 or early S phase (data not shown), nor were we able to rescue the ME2 cell cycle phenotype (decreased proliferation) by supplementation with uridine and cytidine (data not shown).

## Discussion

We have provided the first evidence that the malic enzyme family member ME2 is important in tumor biology; in particular, in the differentiation program of K562 cells. A striking phenotype that we observed upon ME2 knockdown, namely the induction of erythroid differentiation and the inhibition of tumor growth, has previously been observed by other manipulations that affect metabolic pathways in K562 cells. For example, inhibition of BCR-ABL signaling by treatment with imatinib in K562 cells [32] would be expected to decrease glycolysis. K562 differentiation along the erythroid lineage has also been noted after silencing of ATP citrate lyase [33], a key enzyme in lipid synthesis. Another study suggests that ME2 may play an important role in acute lymphoblastic leukemia. French et al. [34] studied the genomic determinants of methotrexate polyglutamates (MTXPGs) variation in acute lymphoblastic leukemia (ALL), and found that ME2 is among 7 genes which displayed all 3 types of genomic variation associated with MTXPGs.

The mechanism by which depletion of ME2 causes tumor cell differentiation remains to be elucidated. One possibility is that malate accumulation plays a role, though our metabolite analysis of whole cell extracts does not appear to support this, since malate levels showed no change in total (mitochondrial plus cytosolic) amount in ME2 knockdown cells as compared with control cells (Table S1). It is conceivable though that mitochondrial malate may be increased in the ME2 knockdown cells. Malate enters the mitochondria from the cytosol (via the malate-aspartate shuttle) [35]. Since the function of ME2 is to convert malate to pyruvate, it is reasonable to assume that the accumulation of malate in mitochondria may be playing a mechanistic role. Supplementation of exogenous malate failed to induce K562 differentiation (data not shown), suggesting that accumulation of this metabolite alone cannot drive differentiation. However, we cannot rule this possibility either, since malate sodium may not be able to get into the cell and into mitochondria. Therefore, it is still possible that mitochondrial malate accumulation explains some of the ME2 knockdown phenotype.

A second possibility is that NADH depletion explains our findings. Mitochondrial reducing equivalent shuttles are critical in regulating the balance in NAD^+^:NADH levels between the cytoplasm and the mitochondria. The resultant effect of shuttle activity is the net transfer of NADH across the inner mitochondria membrane, in the production of ATP and the regeneration of NAD^+^ in the cytoplasm enabling further glucose metabolism and lactate production. The increased drive to accumulate malate in mitochondria due to ME2 silencing may prevent the transferring of malate from cytosol to mitochondria, and render less effective the function of the malate-aspartate shuttle from transferring reduced equivalents from extra-mitochondrial to intra-mitochondrial compartments. Our metabolite data showing decreased NADH/NAD^+^ ratio in ME2 knockdown cells is consistent with this view. Disruption of MAS has been reported to impair viability and fetal growth in mouse blastocysts [36]. In our current investigation we have shown that AOA, an MAS inhibitor, cannot induce K562 differentiation. Instead, we found that AOA induces cell death: apoptosis in ME2 knockdown cells and necrosis in the control cells. The mechanism by which AOA plays different roles in control and knockdown ME2 cells remains to be investigated: it is possible that ATP levels differ under these circumstances. The cause of cell death from AOA is likely due to inhibition of the conversion of glutamate to α-KG, since α-KG rescued the cell death effect.

A third possibility is that pyruvate depletion by ME2 silencing plays a role; however, our metabolite data, given that it is whole cell lysate data, does not definitively address this hypothesis.

We noted that the tumorigenic potential of ME2 deficiency K562 cells *in vivo* was completely absent since injection of those cells into nude mice failed to generate solid tumors. This is intriguing in light of the fact the K562 cells that are fully differentiated as a result of ME2 knockdown in vitro are still growing albeit at a slower rate. It is possible that the knockdown of ME2 indirectly disrupts additional unidentified pathways that are of functional relevance in vivo e.g. critical for establishing tumor take but not manifest in vitro.

Increased ROS generation is an important phenotype of highly glycolytic cancer cells. ROS levels in cancer cells is also to be regulated by the oncogenes such as *Ras, Myc* and *Bcr-Abl* [18], [19], [20] and the tumor suppressor p53 [37]. Many anticancer drugs enhance ROS production and thereby damage cell integrity. However, the manipulation of cellular levels of ROS (smaller increases in ROS than what are needed to cause cell death) can induce cancer cell differentiation [17], [21], [22], [23]. Our studies indicate that silencing of ME2 in K562 cells is accompanied by enhanced ROS generation and increased NAD^+^/NADH and NADP^+^/NADPH ratios. Surprisingly, inhibition of ROS generation by treatment with the antioxidant N-acetyl-cysteine (NAC) could not reverse this differentiation in ME2 knockdown cells, demonstrating that although ROS is produced during K562 differentiation, ROS removal alone was insufficient to inhibit differentiation of K562 cells. The decrease in ATP by about 50%, perhaps due to decreased availability of NADH, may be a contributing factor. In addition it is conceivable the reducing power of NADH may lead to diminished capacity to maintain a high enough ratio of NADPH/NADP^+^, thus compromising, reductive biosynthesis of fatty acids and cholesterol as well as nucleic acid synthesis, needed to fuel cell proliferation. Indeed this sequence of events may account for the increase in ROS that results from depletion of ME2. Moreover, in K562 cells, depletion of ME2 induced K562 erythroid differentiation is accompanied by hemoglobin synthesis. Heme synthesis, which is localized to the mitochondrial matrix, is accompanied by ROS production. In the case of K562 cells, this may explain why we observed ROS levels increasing in the ME2 knockdown cells [38].

There may also be a link between glutamine usage and ME2. In order to obtain energy and the essential precursors for the synthesis of macromolecules, tumor mitochondria adapt by overexpressing glutaminase facilitating the use of glutamate as a fuel. High levels of ME2 can then allow pyruvate to be produced from malate within the mitochondria [2], [9], [39], [40], [41]. ME2 is one of the few progression-linked enzymes in Morris hepatoma series [9], as is a phosphate-dependent glutaminase [42], which strongly suggests an important role of ME2 in glutamine metabolism in tumors [2]. The oxidation of glutamate by tumor mitochondria is accompanied by metabolic interactions with cytosolic malate and/or pyruvate, and extrusion of citrate and alanine into the cytoplasm. These two major products play an important role in tumor metabolism. Citrate is required for fatty acid and cholesterol biosynthesis [43], [44], the latter believed to be characteristically enhanced in tumor cells [45]. Recently, Hatzivassiliou and colleagues found that knockdown of ATP citrate lyase (ACL) also induces K562 cell differentiation [33]. Acetyl-CoA is an important component of fatty acid and cholesterol biosynthesis in the cytosol, since it is the primary enzyme responsible for the synthesis of cytosolic acetyl-CoA which is important in lipid biosynthesis. Hence inhibition of ACL leads to the disruption of lipid synthesis. These results suggest that stimulating cytosolic acetyl-CoA production and lipid synthesis may contribute to the suppression of tumor cell differentiation [46]. Perturbation of the transition of TCA-to-lipid flux in the *in vivo* tumor microenvironment may allow a tumor cell to initiate an adaptive response such as differentiation in order to maintain its survival. The upregulation of specific metabolic intermediates may serve as signals to orchestrate these events. It is possible that citrate may work as an import molecule in this molecular switch. Therefore, disrupting citrate synthesis and transport to cytosol could be an important therapeutic window for targeted tumor therapy. The fact that ME2 selectively uses extra-mitochondria malate to synthesize pyruvate and export citrate for lipid synthesis [2] suggests that malic enzyme might be ideal for this type of targeting. Our results indicated that depletion of ME2 suppresses K562 proliferation and induces differentiation in a manner similar to ACL knockdown. The differentiation induced by knockdown of ME2 and ACL could therefore have a common mechanism, namely inhibition of lipid synthesis. However, our metabolomic analysis (not shown in detail) does not lend support to the idea that ME2 knockdown leads to diminished overall lipid synthesis though we have not made a formal measurement of lipid flux. We did observe significant *shifts* in lipid metabolism. Specifically, there were increases in polyunsaturated free fatty acids and lipids that contain polyunsaturated acyl groups, and decreases in lipids containing saturated lipids. This effect may be driven by the high levels of orotate in the knockdown cells and not directly by ME2 knockdown [47]. Orotate is an important precursor for pyrimidine de novo synthesis. The accumulation of orotate in ME2 depletion cells suggests that pyrimidine metabolism may be blocked. Some data suggests that inhibition of early de novo purine biosynthesis, or specific inhibition of de novo guanine nucleotide biosynthesis, may be an obligatory step in the initiation of differentiation in HL60 and K562 cells [48], [49] induced by certain drugs . Our data suggests that pyrimdine synthesis may be affected, although we were unable to demonstrate the G1/S arrest phenotype that might be expected if this were the mechanism involved in decreased cell proliferation observed in ME2 knockdown cells. Our data indicate that although the ME2 knockdown slowed cell growth, the cells did not accumulate in any one position in the cell cycle and in fact, supplementation with cytidine or uridine could not rescue the differentiation mediated by ME2 depletion (data not shown). Also, the reason why depletion of ME2 leads to the accumulation of orotate is not known, though one target may be the activity or expression of UMP synthase.

Does ME2 depletion affect other cancer cell types? We have found marked effects in several tumor types in vitro and in vivo: in MCF7 breast cancer cells and A549 non-small cell lung cancer cells, ME2 silencing leads to differentiation and to increased apoptosis (data not shown).

K562 cells behave as pluripotent hematopoietic precursor cells [50], [51]. Studies have shown that drugs such as imatinib, butyrate, SB202190, hemin, hydroxyurea and Ara-C induce erythroid differentiation [52], [53], [54], [55], [56], [57], whereas phorbol esters such as PMA induce megakaryocytic differentiation [52]. Both differentiation pathways are driven by the activation of different signal transduction pathways. For megakaryocytic differentiation, cells require the activation of the ERK signaling pathway and the inhibition of p38 MAP kinase [58]. For erythroid differentiation, ERK1/2 signaling inhibition and AKT signaling pathway and GATA-1 transcription factor activation are necessary [55]. In our studies we found ERK1/2 signaling to be inhibited, while AKT signaling was activated in ME2 knockdown K562 cells, consistent with our observed phenotype of erythroid differentiation. At what point in the signaling cascades ME2 knockdown has its effects is under investigation.

In conclusion our data indicate that ME2 plays a crucial role in modulating K562 cell differentiation and growth and highlight a novel role for ME2 as a potentially attractive target for tumor therapy.

## Materials and Methods

### Materials

Dichlorodihydrofluorescein diacetate (CM-H_2_DCF-DA), MitoSOX™, and nonyl acridine orange (NAO) were purchased from Invitrogen/Molecular Probes (Carlsbad, CA). Lipofectamine 2000 and tissue culture reagents were purchased from Invitrogen. The pLKO constructs containing short hairpins RNAi (shRNA) targeted to ME2 and ATP citrate lyase (ACL), and its control vector were purchased from OPEN biosystems. All restriction enzymes were obtained from New England Biolabs. The QIAprep kit was from QIAGEN. Fetal bovine serum (FBS) was obtained from GIBCO. Puromycin, anti-ME2 polyclonal antibody and anti-β-tubulin monoclonal antibody were purchased from Sigma. The anti-vimentin monoclonal antibody was from Santa Cruz Biotechnology. The anti-GATA-1, phospho-ERK1/2, phospho-AKT308, phospho-AKT473 and AKT1/2 polyclonal antibodies were purchased from Cell Signaling Technology. The ERK1/2 moloclonal antibody was from Zymed. The CD235a-FITC and CD10-FITC monoclonal antibodies were obtained from DAKO. Secondary antibodies for enhanced chemiluminescence (ECL) detection were from Amersham Biosciences. All other reagents were of standard analytical grade.

### Cell Culture

The human erythroleukemia K562 cell line was obtained from American Type Culture Collection and grown in Iscove's Modified Medium. All media were supplemented with 10% (v/v) fetal calf serum, 100 units penicillin and 100 µg/ml streptomycin, and grown at 37°C and 5% CO_2_. Cells infected with shRNA virus were selected with 1.0 µg/ml puromycin and stable knock-down of ME2 or ACL were used for analysis.

### Generation of ME2 deficiency cell lines

K562 cells were transduced separately with empty shRNA vector control, three different ME2 and one ACL shRNA lentiviral particles as previously described [59]. ATP citrate lyase (ACL) shRNA lentiviral knockdown cells were used as a positive controls. The three ME2 shRNA sequence (sense) used in this study are: shME2-1, 5′-CGGCATATTAGTGACAGTGTT-3′; shME2-2, 5′-CCCAGTATGGACACATCTTT A-3′; and shME2-3, 5′-GCACGGCTGAAGAA GCATAT A-3′. The shACL sequence is: 5′-GCCTCTTCAATTTCTACGAGGACTT-3′. To produce recombinant lentiviral particles, subconfluent 293FT cells were cotransfected with 3 µg of a shRNA plasmid, and 9 µg virapower packaging mix (Invitrogen) using lipofectamine 2000 (Invitrogen). After 16 h culture medium was switched to regular growth medium and cells were allowed to incubate for additional 48 hours. Conditioned cell culture media containing recombinant lentiviral particles were harvested and frozen. K562 cells were transduced with above cell culture supernatant containing lentiviral particles for 24 hours. These cells were then selected in puromycin (Sigma Aldrich) to generate stable cell lines encoding empty vector shRNA, ME2 shRNA or ACL shRNA. Hereafter, we named those pools pLKO, shME2-1, shME2-2, shME2-3 and shACL, respectively. In order to generate single ME2 knockdown clone, cells from the stable knockdown pools were serially diluted in 96-well plates. The single clone corresponding to its parental pools were named pLKO-s, shME2-1s, shME2-2s and shME2-3s, respectively.

### Western Blotting

K562 cells with and without ME2 knockdown treated with 10 µM LY294002 or DMSO were lysed with RIPA buffer (50 mM Tris-HCl, pH 7.4, 150 mM NaCl, 1% NP-40, 0.1% SDS and 0.5% sodium deoxycholate), and equal amount of proteins were resolved by 4%–12% Bis-Tris gels (Invitrogen), as previously described [60]. Briefly, the proteins were transferred to a PVDF membrane, and membranes were blocked with BLOTTO (5% nonfat dry milk and 0.1% Tween 20 in PBS), and incubated with antisera generated against ME2, phospho-AKT380, phosphor-473, AKT1/2, phosphor-ERK1/2, ERK1, Vimentin, GATA-1 or β-tubulin antibodies respectively. Membranes were washed in PBS plus 0.1% Tween 20 and probed with anti-rabbit or anti-mouse HRP-conjugated secondary antibody (both at 1∶10,000 dilution), and proteins were detected using the ECL Plus chemiluminescence detection reagent (Amersham Biosciences).

### Proliferation assay

Control and ME2 deficient cell lines were plated in 6-well plate at a density of 1×10^5^ cells/well and maintained at 37°C in a 5% CO_2_ incubator. After 24, 72, 120 and 168 hours of initial plating, 0.5 ml cells were diluted into 10 ml of Hanks' buffer and counted by Coulter counter. All samples were assayed in triplicate to generate proliferation curves as described [61].

### Erythroid differentiation assay

Induction of surface expression of the erythroid marker glycophorin A and CD10 was determined by indirect immunofluorescence and flow cytometry as described previously by Hatzivassiliou and colleagues [33]. In brief, K562 cells with or without ME2 knockdown were stained with a mouse FITC-conjugated anti-glycophorin A or CD10 antibody (Dako) at 1∶100 dilution in medium plus 10% FCS for 30 min at 4°C. Control, cells were stained with FITC-conjugated IgG. Cells expressing hemoglobin were determined by benzidine staining as previously described by Park and colleagues [62]. The benzidine dihydrochloride stock solution contained 0.2% w/v benzidine hydrochloride in 3% acetic acid. Cells (1×10^5^) were washed twice in ice-cold PBS. The cell pellets were resuspended in ice-clod PBS. Before staining, 5 µl of 30% H_2_0_2_ was added to 1 ml of a stock solution of benzidine solution. The cell suspensions were mixed with the benzidine solution in a 1∶1 ratio and counted in a hemocytometer after 30 min. Blue cells were considered to be positive for hemoglobin. The proportion of blue-stained cells was quantified under light microscopy. A total of 500 cells were counted for each sample in triplicate. Shown is the mean ± SD of a representative experiment.

### Annexin-V apoptosis assay

Apoptosis was measured by staining with the Nexin reagent using a Nexin kit and counting on the Guava PCA-96 system (Guava Technologies) as per the manufacture's protocol. Briefly, cells were harvested and re-suspended in 100 µl of 1X Nexin buffer, and then mixed with 100 µl of Annexin-V-PE, and Nexin 7-AAD. The cells were allowed to incubate for 20 minutes at room temperature and analyzed in the Guava flowcytometer.

### Determination of cellular reactive oxygen species (ROS)

Intrcellular ROS production was measured by staining with CM-H_2_DCFDA. CM-H_2_DCFDA is a cell-permeant indicator for ROS that is nonfluorescent until removal of the acetate groups by intracellular esterases and oxidation occurs within the cell. The procedure for measuring ROS was carried out as described earlier, with minor modification [63]. Briefly, K562 cells transduced with shRNA lentiviral particles or control vector were selected with puromicin for 2 weeks, and then incubated with 5 µM CM-H_2_DCF-DA for 3 hours, followed by flow cytometry using a FACSCalibur equipped with CellQuest Pro software. Superoxide radicals (O_2_^−^) were measured separately using the MitoSOX reagent according to the manufacturer's protocol (Invitrogen). In brief, cells with or without ME2 knockdown were incubated with 5 µM MitoSOX™ reagent for 10 minutes at 37°C, then washed three times and observed under a fluorescence microscope using the Rhodamine filter and Axiovision software for capturing images (Zeiss, Germany).

### Determination of oxidative damage to mitochondrial membranes

Mitochondrial membrane lipid peroxidation was detected as described [63]. K562 cells transduced with shRNA lentiviral particles or control vector were selected with puromycin for 2 weeks, and then labeled with 50 nM NAO for 20 min and analyzed by flow cytometry using FL2 or 3 filters and Cell Quest software analysis data (Becton Dickson).

### Xenograft model in nude mice

Animal experiments were performed under federal guidelines and approved by the Institutional Animal Care and Use Committee (IACUC) of the Beth Israel Deaconess Medical Center (Approval number 0342007). K562 xenografts in nude mice were generated by following the description of Verrax J et al. [64]. Briefly, approximately 10^7^ ME2 deficient or control K562 cells resuspended in 200 µl of a serum-free culture medium/Matrigel mixture (1∶1) were subcutaneously injected into the right and left flanks of male nude/nu/nu athymic mice, respectively. Tumor-bearing mice were sacrificed after 6–8 weeks and tumor masses were measured or imaged before excision. Tumor lysates were prepared by homogenization of tumor tissues in RIPA lysis buffer and were resolved by SDS-PAGE and transferred onto PDVF membranes and immunoblotted with anti-ME2 antibody and normalized by β-tubulin as a loading control.

### Intracellular ATP measurements

Intracellular ATP levels in control and ME2 deficient cells were measured by ATP Bioluminescence Assay Kit CLS II (Roche, Germany) according to manufacturer's instructions. Briefly, cells were diluted to a concentration of 10^7^ cells/ml, then add 9 volumes of boiling lysis buffer (100 mM Tris, 4 mM EDTA, pH 7.75) and incubated for another 2 minutes at 100°C. Cell lysates were collected by centrifugation and pelleting at 1000× g for 1 min, and 50 µl of samples were transferred into a MTP-well, and mixed with 50 µl luciferase reagent. Luminescence was measured using a luminescence reader (Molecular Devices), and normalized for protein concentration.

### NAD^+^/NADH assay

Intracellular NAD^+^ and NADH levels in control and ME2 deficient cells were measured by NAD^+^/NADH Assay Kit (Abcam, San Francisco, CA) according to manufacturer's instructions. Briefly, 2×10^5^ cells were washed with cold PBS and extacted with NADH/NAD Extraction Buffer by freeze/thaw two cycles (20 min on dry-ice, then 10 min at room temperature). Total NADt and NADH were detected following the instruction in a 96-well plate and color were developed and read at 450 nm. The concentration of NADt or NADH was expressed in pmol/10^6^ cells. NAD/NADH Ratio is calculated as: [NADt – NADH]/NADH.

### Metabolite profiling

To determine differences in metabolite profiles between ME2-depleted and control cells, metabolite extracts were prepared and then analyzed using liquid chromatography tandem mass spectrometry (LC-MS). Briefly, K562 cells with or without ME2 depletion were washed once and resuspended in fresh growth medium and grown an additional 2–4 h. To initiate the extractions, cells were spun down and media were vacuum aspirated. Lipids were extracted with the addition of 4 mL isopropanol (4°C) and polar metabolites were extracted with the addition of 4 mL 80% methanol (−80°C). Following the addition of extraction solvent, the samples were vortexed for 1 minute, held for 1 h (4°C for lipid extracts; −80°C for polar metabolite extracts), and then centrifuged at 3500 rpm for 10 minutes. The supernatants were transferred to new tubes. For polar metabolites, the pellet was resuspended in 1 ml 80% methanol (−80°C), vortexed for another 1 min, centrifuged as described above, and the supernatants were combined.

LC-MS data were acquired using 4000 QTRAP triple quadrupole mass spectrometers (Applied Biosystems/Sciex, Foster City, CA) equipped with HTS PAL autosamplers (Leap Technologies, Carrboro, NC) and either Agilent 1100 Series or Agilent 1200 Series binary HPLC pumps (Santa Clara, CA). Several chromatographic methods were used to profile endogenous metabolites. Lipids were analyzed using a ProSphere C4 HPLC column (150×3 mm; Grace, Columbia, MD) and full scan MS data were acquired in the positive ion mode. Biogenic amines and other positively charged polar metabolites were separated using an Atlantis HILIC column (150×2.1 mm; Waters, Milford, MA) that was eluted with a 10 min linear gradient, initiated with 100% mobile phase B (acetonitrile with 0.1% formic acid, v/v) and concluding with 60% mobile phase A (10 mM ammonium formate and 0.1% formic acid, v/v). Central metabolites, pyrimidines, and other negatively charged polar compounds were analyzed using the ion paring chromatography method described by Lou et al. with minor modification to the gradient program [65]. Mobile phases used in the modified method were 10 mM tributylamine/15 mM acetic acid (mobile phase A) and methanol (mobile phase B) and the column was eluted at a flow rate of 200 µl/min using the following program: 100% mobile phase A at initiation, 100% A at 4.5 min, 80% A at 7.5 min, 70% mobile phase A at 26.5 min, 2% mobile phase A at 36.5 min, and 2% mobile phase A at 40.5 min. Multiple reaction monitoring (MRM) was used to acquire targeted MS data for specific metabolites in the in the positive (HILIC method) and negative ion (ion paring method) modes. Declustering potentials and collision energies were optimized for each metabolite by infusion of reference standards prior to sample analyses. The scheduled MRM algorithm in the Analyst 1.5 software (AB SCIEX; Foster City, CA) was used to automatically set dwell times for each transition. MultiQuant software (Version 1.1; AB SCIEX; Foster City, CA) was used for automated peak integration and metabolite peaks were manually reviewed for quality of integration and compared against a known standard to confirm identity.

## Supporting Information

Figure S1 Stable knockdown of endogenous ME2 levels in K562 cells induces erythroid differentiation. The percentage of hemoglobin-expressing cells in control (pLKO) and ME2 knockdown (shME2-1, shME2-2 and shME2-3) cell populations was determined by benzedrine staining. Plotted is the mean ± SD from triplicate samples from a representative experiment.
(0.12 MB TIF)

Figure S2 The effect of α-ketoglutarate (α-KG) on AOA-induced K562 cell death. K562 cells with or without ME2 knockdown were incubated with 0.1 mM AOA plus 2 mM α-KG for 48 h. Cell death was assessed by flow cytometry. A. pLKO K562 cells without any treatment. B. pLKO K562 cells treated with 0.1 mM AOA for 48 h. C. pLKO K562 cells treated with 0.1 mM AOA combined with 2 mM α-KG. Data are representative of two independent experiments.
(0.32 MB TIF)

Table S1 Metabolite levels in lysates from control K562 (pLKO) and ME2 knockdown (shME2-3) cells. The metabolites were measured by LC-MS method as described in “Materials and Methods”.
(0.05 MB XLS)
