# Supplementary figures and images for: Induction of Erythroid Differentiation in Human Erythroleukemia Cells by Depletion of Malic Enzyme 2
Source: PLoS One. 2010 Sep 2;5(9):e12520. doi: 10.1371/journal.pone.0012520 (PMC2932743; doi:10.1371/journal.pone.0012520)

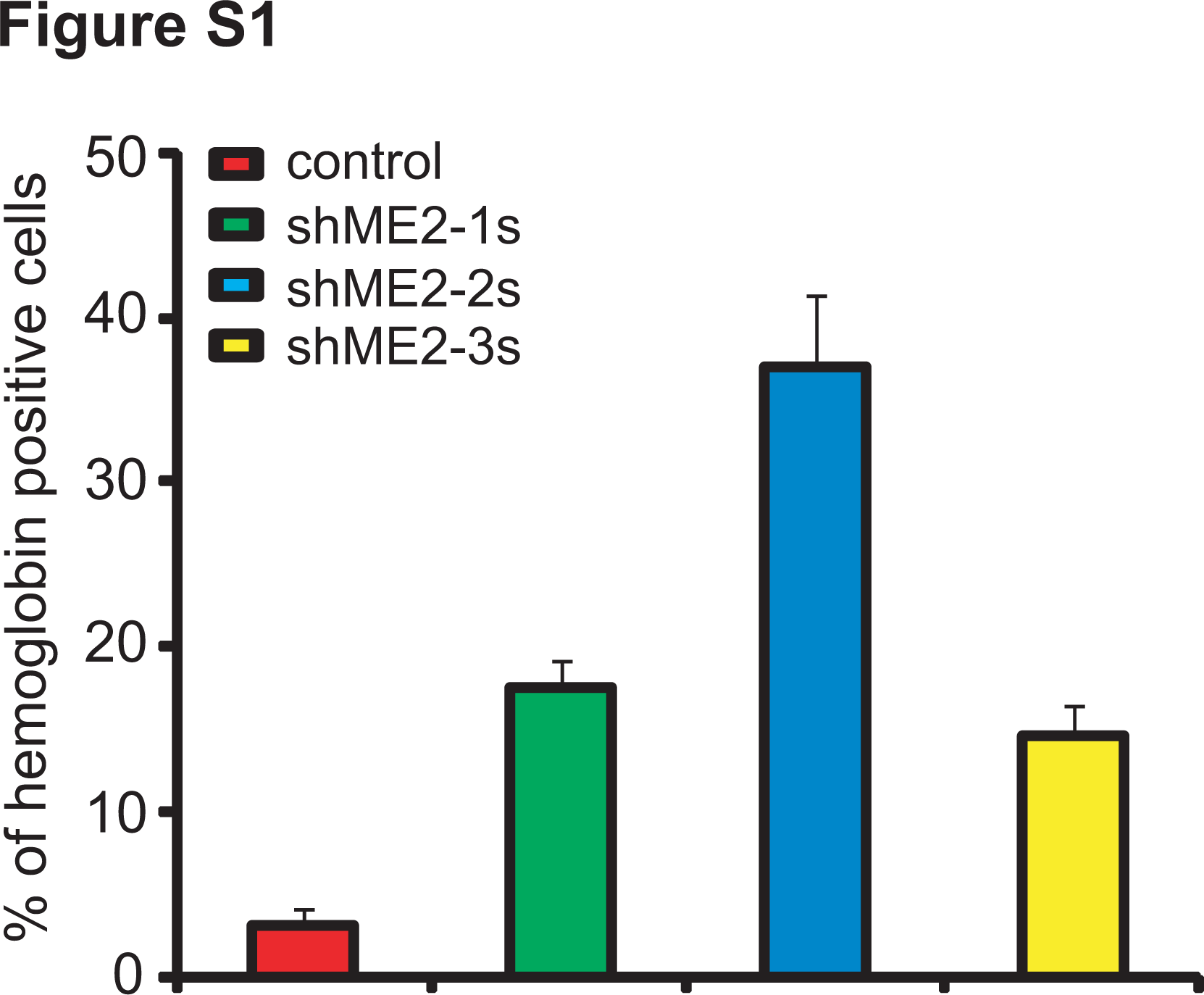

Supplement: Figure S1 — Stable knockdown of endogenous ME2 levels in K562 cells induces erythroid differentiation. The percentage of hemoglobin-expressing cells in control (pLKO) and ME2 knockdown (shME2-1, shME2-2 and shME2-3) cell populations was determined by benzedrine staining. Plotted is the mean ± SD from triplicate samples from a representative experiment. (0.12 MB TIF) [file pone.0012520.s001.tif]

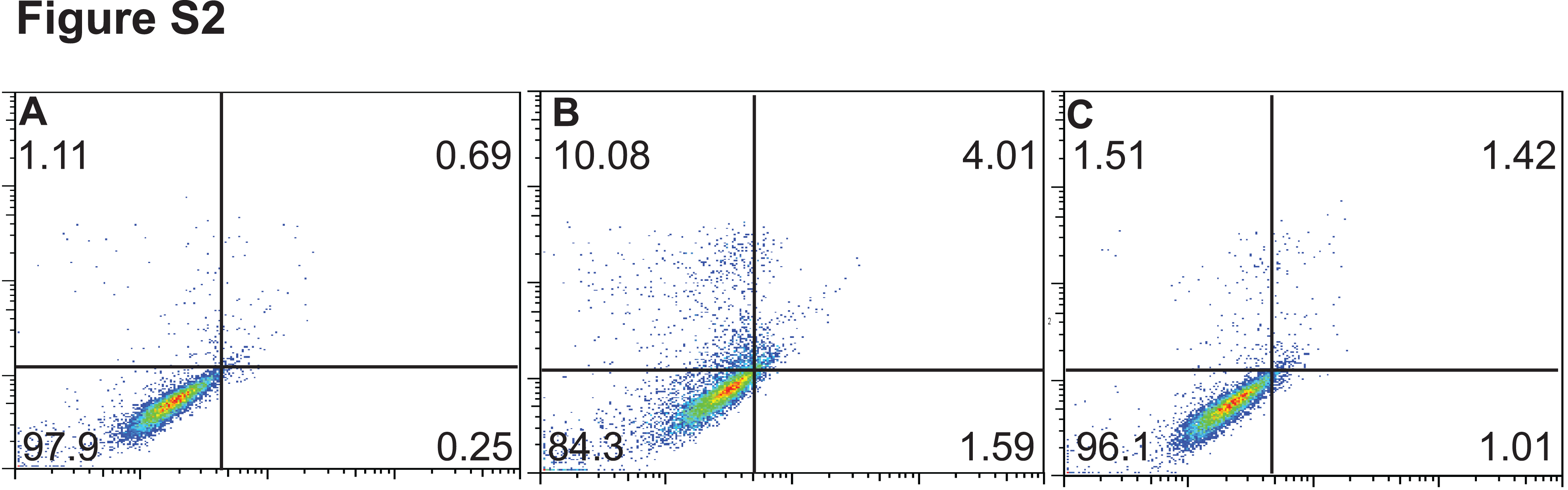

Supplement: Figure S2 — The effect of α-ketoglutarate (α-KG) on AOA-induced K562 cell death. K562 cells with or without ME2 knockdown were incubated with 0.1 mM AOA plus 2 mM α-KG for 48 h. Cell death was assessed by flow cytometry. A. pLKO K562 cells without any treatment. B. pLKO K562 cells treated with 0.1 mM AOA for 48 h. C. pLKO K562 cells treated with 0.1 mM AOA combined with 2 mM α-KG. Data are representative of two independent experiments. (0.32 MB TIF) [file pone.0012520.s002.tif]
